# ZipA Binds to FtsZ with High Affinity and Enhances the Stability of FtsZ Protofilaments

**DOI:** 10.1371/journal.pone.0028262

**Published:** 2011-12-02

**Authors:** Anuradha Kuchibhatla, Anusri Bhattacharya, Dulal Panda

**Affiliations:** Wadhwani Research Center, Department of Biosciences & Bioengineering, Indian Institute of Technology Bombay, Mumbai, India; Russian Academy of Sciences, Institute for Biological Instrumentation, Russian Federation

## Abstract

A bacterial membrane protein ZipA that tethers FtsZ to the membrane is known to promote FtsZ assembly. In this study, the binding of ZipA to FtsZ was monitored using fluorescence spectroscopy. ZipA was found to bind to FtsZ with high affinities at three different (6.0, 6.8 and 8.0) pHs, albeit the binding affinity decreased with increasing pH. Further, thick bundles of FtsZ protofilaments were observed in the presence of ZipA under the pH conditions used in this study indicating that ZipA can promote FtsZ assembly and stabilize FtsZ polymers under unfavorable conditions. Bis-ANS, a hydrophobic probe, decreased the interaction of FtsZ and ZipA indicating that the interaction between FtsZ and ZipA is hydrophobic in nature. ZipA prevented the dilution induced disassembly of FtsZ polymers suggesting that it stabilizes FtsZ protofilaments. Fluorescein isothiocyanate-labeled ZipA was found to be uniformly distributed along the length of the FtsZ protofilaments indicating that ZipA stabilizes FtsZ protofilaments by cross-linking them.

## Introduction

FtsZ is a homolog of the eukaryotic cell division protein tubulin and it plays an essential role in bacterial cell division [1]–[4]. FtsZ monomers polymerize to form a polymeric ring structure, called the Z-ring in bacteria [5]. The Z-ring is a dynamic structure that undergoes assembly and disassembly during the period of cell division [6]. The assembly dynamics of FtsZ is regulated in cooperation with two antagonistic factors. A group of proteins is known to enhance FtsZ assembly *in vitro* and to stabilize the Z-ring in bacteria while another group of proteins has been shown to inhibit FtsZ assembly *in vitro* and to destabilize the Z-ring in bacteria [7]–[9].

ZipA is an early cell division protein, which is known to interact with FtsZ directly [10], [11]. It enhances the FtsZ assembly and bundling *in vitro* [12], [13]. The C-terminus of FtsZ binds to the C-terminus of ZipA [13], [14]. Since the interaction between FtsZ and ZipA is essential for cell division, several inhibitors were discovered with an idea that these inhibitors may have antibacterial potential [15]–[18]. Impeding the FtsZ-ZipA interaction was found to block the cell division leading to long filamentous bacteria and consequently bacterial cell death [16]. Thus, the inhibition of interaction between FtsZ and ZipA has become an important strategy for finding new class of antibacterial drugs.

In this study, we have determined the equilibrium binding of ZipA with FtsZ using fluorescence spectroscopy, which may be used to identify inhibitors of ZipA-FtsZ interaction. The assembly and bundling of FtsZ have been found to decrease strongly with increasing pH from 6.0 to 7.9 [19], [20]. The intracellular pH of *E. coli* was estimated to be 7.4–7.8 [21]. Since alkaline pH strongly inhibits the assembly of FtsZ *in vitro* [19] and the intracellular pH of *E. coli* is 7.4–7.8, it is logical to think that some cellular factors/proteins may assist in the efficient assembly of FtsZ in *E. coli*. We therfore examined the effect of ZipA on the assembly of FtsZ at higher pHs and found that ZipA supports efficient assembly of FtsZ at pH 8.0. We have also provided evidence indicating that hydrophobic interactions play an important role in the binding of ZipA and FtsZ and that ZipA stabilizes FtsZ protofilaments by binding along its length.

## Materials and Methods

### Materials

Fluorescein isothiocyanate (FITC), Piperazine-1,4-bis(2-ethanesulfonic acid) (PIPES), isopropyl-β-D-thiogalactopyranoside (IPTG), guanosine 5′-triphosphate sodium salt hydrate (GTP), ethylenediaminetetraacetic acid (EDTA), phenylmethylsulfonyl fluoride (PMSF), β-mercaptoethanol (β-ME), lysozyme, bovine serum albumin (BSA) and Tris-HCl were purchased from Sigma. 4,4′-dianilino-1,1′-binaphthyl-5,5′-disulfonic acid (bis-ANS) was purchased from Molecular Probes. Bio-Gel P6 resin was procured from Bio-Rad. Ni-NTA was obtained from Qiagen.

### Purification of FtsZ

The recombinant *E. coli* FtsZ was overexpressed and purified from *E.coli* BL21 strain as described previously [22]. Briefly, the cells were grown at 37°C to OD_600_ of 0.6, induced with 0.4 mM IPTG and harvested after 4 h. The cells were suspended in lysis buffer [50 mM Tris, pH 8.0, 100 mM NaCl, 1 mM ethylenediaminetetraacetic acid (EDTA), 1 mM phenylmethylsulfonyl fluoride (PMSF), 0.1% β-mercaptoethanol (β-ME) and 1 mM MgCl_2_] on ice. Subsequently, the cell lysate was subjected to ammonium sulfate precipitation. The salt was removed using a desalting column (Bio-Gel P6 resin, Bio-Rad) equilibrated with 25 mM PIPES, pH 6.8. Then, the protein was further purified using a polymerization and depolymerization step [22]. FtsZ concentration was determined by Bradford method using bovine serum albumin (BSA) as a standard [23]. The purified protein was aliquoted and stored frozen at −80°C.

### Purification of ZipA

Recombinant *E. coli* His-ZipA (a kind gift from Dr. D. RayChaudhuri of Tufts University School of Medicine) was over expressed and purified from *E.coli* BL21(DE3) strain with ampicillin in the medium. The cells were grown at 37°C to OD_600_ of 0.8, induced with 1 mM IPTG and harvested after 5 h. The harvested culture was lysed under denaturing conditions using 8 M urea in extraction buffer containing 50 mM Tris, 200 mM NaCl, pH 8.0. Lysozyme (0.4 mg/ml) was added to cell suspension and was then incubated on ice for 1 h. Partially lysed cells were sonicated for ten cycles with 30 sec pulses. Then, the lysed cells were spun to obtain a clear supernatant. The supernatant was incubated with Nickel-NTA beads for 90 min at 4°C to allow binding of the overexpressed His-ZipA recombinant protein. After adding the cell lysate to the nickel column, the column was washed extensively with 50 mM Tris buffer, pH 8.0 containing 150 mM NaCl (TN buffer). The beads were washed with TN buffer with increasing concentrations of imidazole. ZipA was eluted at 150 mM imidazole concentration. The protein was passed through a P-6 column (Bio-Gel P6 resin, Bio-Rad) equilibrated with 50 mM Tris buffer (pH 8.0) to desalt the protein. The protein was eluted with 50 mM Tris buffer (pH 8.0). The protein purity was checked with SDS-PAGE and a single band was obtained. The ZipA concentration was determined by Bradford's method using BSA as a standard [23]. ZipA was stored at −80°C.

### Light scattering assay

FtsZ (6 µM) in buffer containing 25 mM PIPES, pH 6.8 and 1 mM MgCl_2_ was incubated for 10 min in the absence and presence of 2 and 4 µM ZipA on ice. After the addition of 1 mM GTP, the assembly kinetics was monitored at 37°C by light scattering (400 nm) using a JASCO 6500 fluorescence spectrophotometer (Tokyo, Japan).

In another experiment, FtsZ (6 µM) in 25 mM PIPES buffer, pH 6.8, containing 1 mM MgCl_2_ and 1 mM GTP was transferred to 37°C and the assembly was monitored for 5 min. Then, 4 µM of ZipA was added to the assembly mixture and the assembly kinetics was monitored for an additional 10 min. Light scattering signals were corrected by subtracting the appropriate blanks.

### Dilution induced disassembly assay

FtsZ (25 µM) in 25 mM PIPES (pH 6.8), 1 M glutamate, 0.1 mM MgCl_2_ and 1 mM GTP was polymerized for 10 min. The polymers formed were diluted 20 times with warm 25 mM PIPES buffer in the absence and presence of 1, 2, 3 and 4 µM ZipA. The final concentration of FtsZ after dilution was 1.25 µM. The diluted polymeric suspension was incubated for an additional 5 min. The polymers were then centrifuged at 88,760× g for 30 min at 30°C. Pellets were dissolved in SDS-containing buffer. The samples were analyzed by Coomassie-blue stained SDS-PAGE. The band intensity was analyzed using Image J Pro Plus software.

### Fluorescence Microscopy

ZipA possesses several lysine residues, which can be covalently modified using FITC. ZipA and FITC were incubated in the ratio of 1∶5 in 50 mM sodium phosphate buffer, pH 8.0 for 4 h on ice. Then, the protein was centrifuged at a speed of 88,760× g for 10 min to remove any aggregate formed during the incubation time. Unbound FITC was removed from that of the bound FITC-ZipA by a gel filtration (Bio-Gel P6 resin Bio-Rad) column, which was pre-equilibrated with 20 mM Tris buffer (pH 8.0). The concentration of FITC bound to ZipA was determined from the absorbance at 495 nm using a molar extinction coefficient of 77,000 M^−1^cm^−1^ and the concentration of ZipA was determined by the method of Bradford [23]. The incorporation ratio of FITC per ZipA molecule was determined to be 0.6. A similiar procedure was followed to label FtsZ by FITC.

FtsZ (2 µM) in buffer containing 25 mM PIPES, pH 6.8 and 1 mM MgCl_2_ was incubated for 10 min in the absence and presence of different concentrations (0.5, 1.0 and 2.0 µM) of FITC-ZipA. To this mixture, 1 mM GTP was added and FtsZ was polymerized at 37°C for 10 min. The sample was placed on a cover slip, which was inverted on a glass slide and observed using a fluorescence microscope (Nikon ECLIPSE TE2000-U). The images were captured using a CoolSNAP-Pro camera. A similiar procedure was followed to determine the effect of ZipA on the assembly of FITC labeled FtsZ (FITC-FtsZ).

### Fluorescence spectroscopy

FITC-ZipA (0.5 µM) was incubated without or with different concentrations (10–2000 nM) of FtsZ in 50 mM PIPES buffer of different pHs (6.0, 6.8 and 8.0) for 10 min at 25°C. The fluorescence spectra (510–550 nm) of the samples were recorded using 495 nm as the excitation wavelength. The increase in the FITC-ZipA fluorescence at 518 nm upon binding to FtsZ was used to determine the dissociation constant (K_d_) of the interaction of FITC-ZipA and FtsZ using the equation:
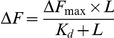
Where, ΔF is the change in the fluorescence intensity of the FITC-ZipA upon binding to FtsZ, ΔF_max_ is the maximum change in the fluorescence intensity of FITC-ZipA when it is bound with FtsZ and L is the concentration of FtsZ. The ΔF_max_ value was calculated using the GraphPad Prism 5 software. ΔF was calculated by subtracting the fluorescence intensity of FITC-ZipA in the absence of FtsZ from the fluorescence intensity of FITC-ZipA in the presence of FtsZ. The data were analyzed using GraphPad Prism 5 software.

### Sedimentation assay

FtsZ (6 µM) in 50 mM PIPES buffer of different (6.0, 6.8 and 8.0) pHs was incubated for 10 min in the absence and presence of 2 µM ZipA. Then, 1 mM MgCl_2_ and 1 mM GTP were added to the reaction mixture and polymerized for 10 min at 37°C. FtsZ polymers were sedimented at 88,760× g at 30°C for 30 min and pellets were dissolved in SDS-containing buffer. The samples were analyzed by Coomassie-blue stained 10% SDS-PAGE. The band intensity was analyzed using Image J Pro Plus software. To examine whether bis-ANS had an adverse effect on the interaction of ZipA and FtsZ, FtsZ (6 µM) in 25 mM PIPES (pH 6.8), 50 mM KCl and 1 mM MgCl_2_ was incubated without and with different concentrations of bis-ANS on ice for 10 min. Then, ZipA (4 µM) was added to each of the reaction mixtures and incubated for an additional 5 min on ice. Subsequently, 1 mM GTP was added to the reaction mixtures and polymerized for 10 min at 37°C. FtsZ polymers were sedimented at 88,760× g at 30°C for 30 min. The pellets were resuspended in SDS-containing buffer and the amount of FtsZ in the pellets was estimated by Coomassie-blue staining of the 10% SDS-PAGE.

### Electron Microscopy

FtsZ (6 µM) in 50 mM PIPES buffer of different (6.0, 6.8 and 8.0) pHs was incubated for 10 min in the absence and presence of 2 µM ZipA. Then, 1 mM MgCl_2_ and 1 mM GTP were added to the reaction mixtures and polymerized for 10 min at 37°C. The samples were taken on carbon coated grids and negatively stained using uranyl acetate as described earlier [24], [25]. The grids were observed under TECHNAI G^2^ transmission electron microscope. For comparison, 2 µM ZipA in the absence of FtsZ was treated in a similar manner and observed under microscope.

### Effects of bis-ANS on the interaction of FtsZ and FITC-ZipA

FITC-ZipA (0.5 µM) was incubated without and with different concentrations (0.5, 1.0, 2.0 and 4.0 µM) of bis-ANS in 50 mM PIPES, pH 6.8 for 5 min at 25°C. Then 0.5 µM of FtsZ was added to the reaction mixtures and incubated for another 15 min at 25°C. The fluorescence emission at 518 nm was monitored using 495 nm as the excitation wavelength. For all the measurements the respective blanks containing FITC-ZipA and bis-ANS were subtracted and the change in fluorescence intensity was calculated.

## Results

### ZipA enhanced FtsZ assembly *in vitro*

**Figure 1:**
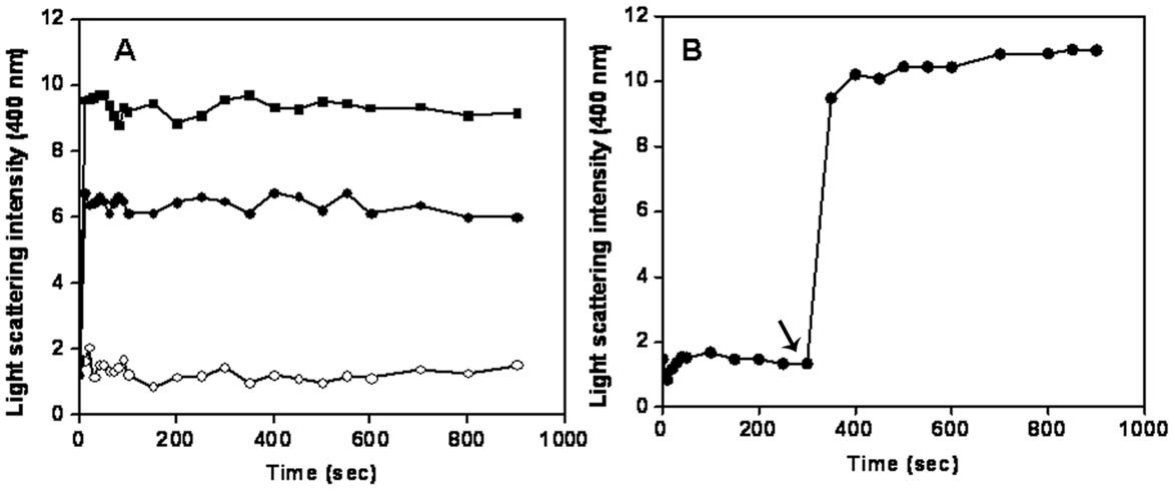
Effects of ZipA on the assembly kinetics of FtsZ. FtsZ (6 µM) was polymerized in the absence (○) and presence of 2 µM (•) and 4 µM (▪) of ZipA (Panel A). FtsZ (6 µM) was polymerized in the absence of ZipA for 5 min. Then, 4 µM ZipA was added to the reaction milieu (indicated by an arrow) and the assembly kinetics of FtsZ was monitored for an additional 10 min (Panel B).

Using sedimentation assay and electron microscopy, it has been shown that ZipA enhances the assembly of FtsZ *in vitro* [12], [13]. Consistent with the previous reports [12], [13], ZipA was found to enhance the light scattering intensity of FtsZ assembly in a concentration dependent fashion indicating that it enhances the assembly and bundling of FtsZ (Fig. 1A). For example, the light scattering intensity was found to increase by 2.5±0.5 and 4.4±0.6 folds in the presence of 2 and 4 µM ZipA, respectively as compared to the control. In a separate experiment, FtsZ was polymerized alone for 5 min; then, 4 µM of ZipA was added to the cuvette and the polymerization reaction was monitored for an additional 10 min (Fig. 1B). The addition of ZipA in the assembly milieu caused a sudden increase in the light scattering signal of the FtsZ assembly supporting the idea that ZipA enhances the assembly and bundling of FtsZ (Fig. 1B).

### ZipA stabilized FtsZ polymers against dilution induced disassembly

**Figure 2:**
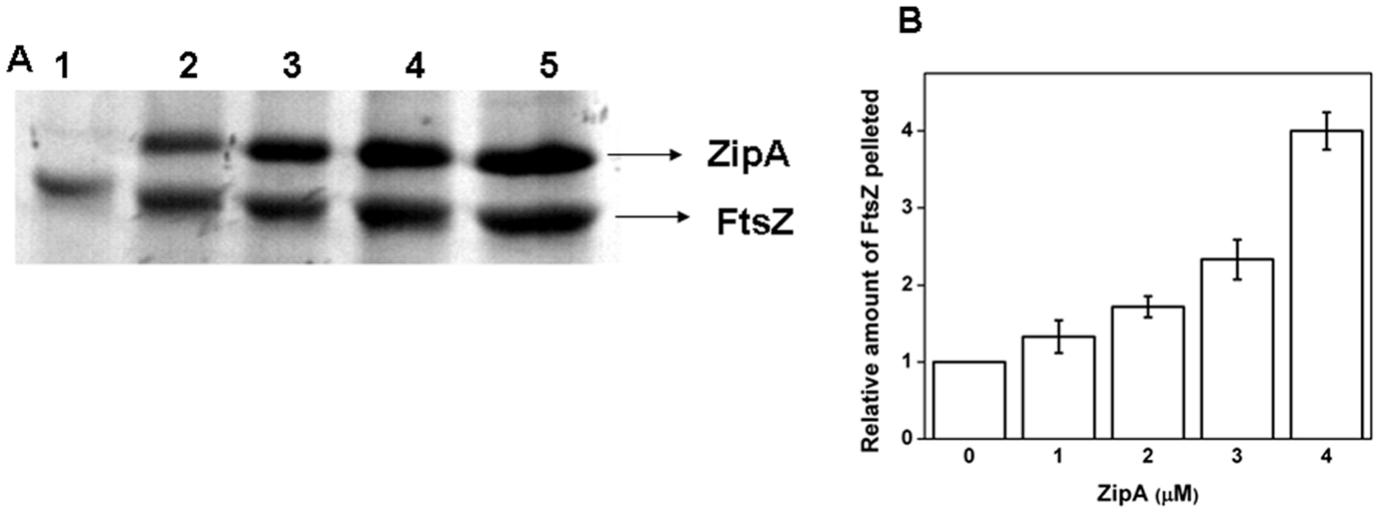
ZipA prevented dilution-induced disassembly of FtsZ polymers. FtsZ (25 µM) was polymerized as described in the materials and methods section. The preformed FtsZ polymers were diluted 20 times in warm 25 mM PIPES buffer, pH 6.8 without and with different concentrations of ZipA and incubated for an additional 5 min at 37°C. The polymers were collected through centrifugation and the amount of FtsZ in the pellet was estimated using Coomassie blue stained SDS-PAGE. Lanes 1–5 denote FtsZ polymers pelleted in the absence and presence of 1, 2, 3 and 4 µM ZipA, respectively (Panel A). The relative amount of FtsZ in the pellets with respect to control was plotted against ZipA concentration (Panel B).

FtsZ monomers assemble to form highly dynamic polymers [26]–[28] and FtsZ polymers are known to disassemble upon dilution [29]–[31]. Therefore; we used the dilution-induced disassembly of FtsZ polymers as a strategy to asses whether ZipA could stabilize FtsZ polymers. Preformed FtsZ polymers were diluted 20 times with warm 25 mM PIPES buffer in the absence and presence of 1, 2, 3 and 4 µM ZipA and incubated for 5 min at 37°C. Then, the polymers were collected through high speed sedimentation. The recovery of polymeric FtsZ was found to increase with increasing concentrations of ZipA (Figs. 2A and B). For example, the amount of FtsZ pelleted was increased by ∼4-fold in the presence of 4 µM ZipA, as compared to the control (Fig. 2B). The result suggested that ZipA prevented the disassembly of FtsZ polymers.

### FITC-ZipA copolymerized with FtsZ and uniformly decorated FtsZ filaments

**Figure 3:**
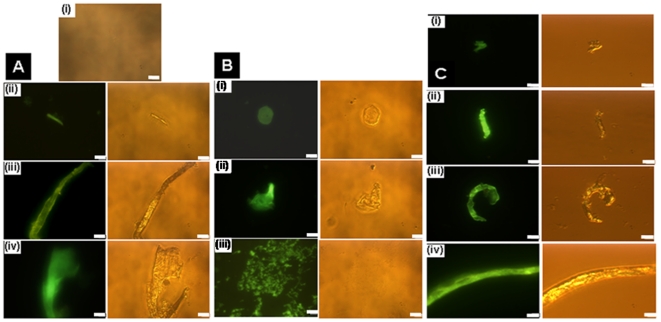
ZipA induced bundling of FtsZ and co-polymerized with FtsZ. FtsZ (2 µM) was polymerized in 25 mM PIPES buffer, pH 6.8 containing 1 mM MgCl_2_ and 1 mM GTP at 37°C in the absence and presence of ZipA. FtsZ polymers were observed using a fluorescence microscope and a differential interference contrast microscope. FtsZ was polymerized in the absence (i), and presence of 0.5 (ii), 1.0 (iii) and 2.0 (iv) µM FITC-ZipA (Panel A). 0.5 (i), 1.0 (ii) and 2.0 (iii) µM of FITC-ZipA in the absence of FtsZ are shown (Panel B). FITC-FtsZ was polymerized in the absence (i), and presence of 0.5 (ii), 1.0 (iii) and 2.0 (iv) µM ZipA (Panel C), respectively. Scale bar is 10 µm.

To examine whether FtsZ could copolymerize with ZipA, FtsZ was polymerized in the absence and presence of different concentrations of FITC-ZipA. The polymer bound FITC-ZipA was visualized by fluorescence microscopy. In the absence of FITC-ZipA, FtsZ formed only few thin polymers (Fig. 3A (i)). In the presence of FITC-ZipA, FtsZ protofilaments were clearly visible (Fig. 3A (ii, iii, iv). Short FtsZ protofilaments were visible in the presence of 0.5 µM FITC-ZipA (Fig. 3A (ii)) and thick bundles of FtsZ were observed at higher concentrations of FITC-ZipA (Fig. 3A (iii and iv)). The images also showed that FITC-ZipA induced the bundling of FtsZ protofilaments. The uniform recruitment of FITC-ZipA in FtsZ bundles suggested that ZipA formed stable complexes with FtsZ. Only FITC-ZipA did not form filamentous polymers instead it formed aggregates (Fig. 3B). To confirm the results of bundling and to rule out the possibility of artifacts, a similar experiment was performed using FITC-FtsZ (Fig. 3C). Consistent with the previous experiment, ZipA was found to induce heavy bundling of FtsZ polymers (Fig. 3C (ii, iii and iv).

### ZipA bound to FtsZ with high affinities at different pHs

**Figure 4:**
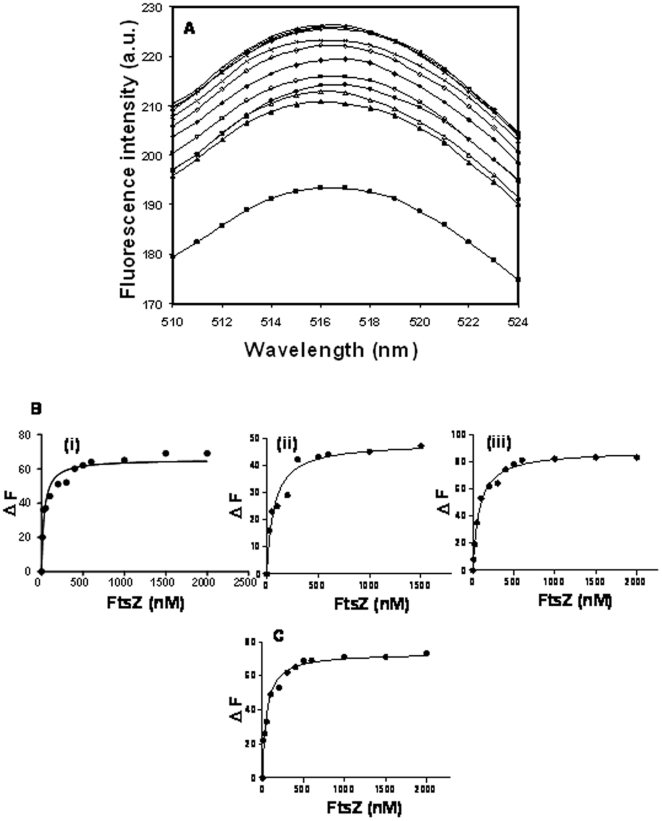
Characterization of equilibrium binding of FITC-ZipA to FtsZ. The fluorescence emission spectra of FITC-ZipA (0.5 µM) at pH 6.8 in the absence (▪) and presence of 10 (▴), 25 (▵), 50 (•), 100 (○), 200 (♦), 300 (◊), 500 (x), 600 (*), 1000 (+), 1500 (−) nM FtsZ (Panel A). Excitation wavelength used was 495 nm. The changes in the fluorescence intensities of FITC-ZipA in the presence of different concentrations of FtsZ at pHs of 6.0 (i), 6.8 (ii) and 8.0 (iii) (Panel B) and in the presence of 500 mM NaCl at pH 6.8 (Panel C) are shown.

FtsZ increased the fluorescence intensity of FITC-ZipA in a concentration dependent manner (Fig. 4A). The dissociation constant (K_d_) of the interaction between ZipA and FtsZ was determined at different pHs. The K_d_ was estimated to be 43±20, 65±6 and 85±10 nM at pHs of 6.0, 6.8 and 8.0, respectively (Fig. 4B (i), (ii) and (iii)) suggesting that the binding affinity of ZipA and FtsZ decreased with increasing pH from pH 6.0 to pH 8.0 (p<0.05). Further, FITC-ZipA was found to bind to FtsZ with a K_d_ of 50±10 nM in the presence of 500 mM NaCl at pH 6.8 (Fig. 4C).

### Effects of ZipA on the assembly of FtsZ *in vitro* at different pHs

**Figure 5:**
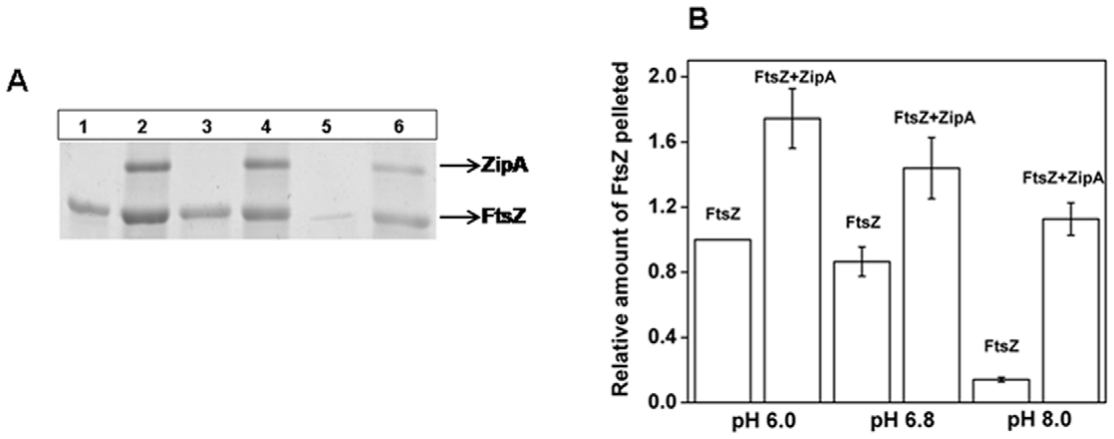
Effects of ZipA on the polymerized amount of FtsZ at different pHs. FtsZ (6 µM) was polymerized in 50 mM PIPES buffer containing 1 mM MgCl_2_ and 1 mM GTP at 37°C in the absence and presence of ZipA (2 µM) at different pHs. FtsZ polymers were collected by centrifugation. Coomassie blue stained SDS-PAGE of the sedimented polymers at pH 6.0 (Lane 1 and 2), pH 6.8 (Lane 3 and 4) and pH 8.0 (Lane 5 and 6) (Panel A). Lanes 1, 3 and 5 are in the absence of ZipA and lanes 2, 4 and 6 are in the presence of ZipA (Panel A). ZipA increased the polymerized amount of FtsZ (Panel B).

FtsZ was polymerized at different pHs (6.0, 6.8 and 8.0) in the absence and presence of ZipA. The polymers were collected through centrifugation. An analysis of the Coomassie blue stained SDS-PAGE showed a decrease in the amount of polymerized FtsZ with increasing pH with or without ZipA (Fig. 5A and B). However, the reduction in the amount of polymeric FtsZ with increasing pH was significantly higher in the absence of ZipA than its presence.

**Figure 6:**
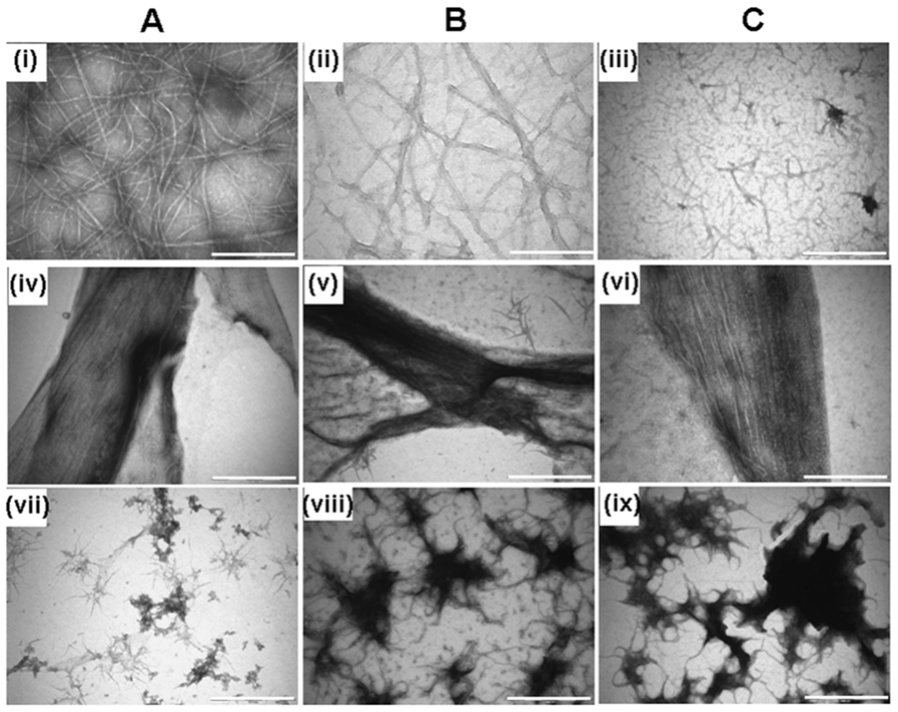
Effects of ZipA on the assembly and bundling of FtsZ at different pHs. FtsZ (6 µM) was polymerized in 50 mM PIPES buffer containing 1 mM MgCl_2_ and 1 mM GTP at 37°C in the absence (i, ii, iii) and presence (iv, v, vi) of ZipA (2 µM) at pHs of 6.0 (Panel A), 6.8 (Panel B) and 8.0 (Panel C). ZipA formed aggregates at different pHs used in this study (vii, viii, ix). Scale bar is 1000 nm.

The effect of pH on the assembly of FtsZ in the absence and presence of ZipA was also examined using electron microscopy (Fig. 6). In the absence of ZipA, FtsZ polymerization decreased substantially with increasing pH (Fig. 6 (i, ii and iii)). At pHs of 6.0 and 6.8, extensive networks of long FtsZ polymers were observed (Fig. 6 (i and ii). However, at pH 8.0, short FtsZ protofilaments sparsely occupied were found (Fig. 6 (iii)). In the presence of ZipA, thick bundles of FtsZ polymers were observed at all pHs examined (Fig. 6 iv–vi). Even at pH 8.0 (FtsZ alone could not polymerize greatly), ZipA could induce thick bundles of FtsZ protofilaments (Fig. 6 vi). However, FtsZ bundles appeared to be loosely packed suggesting that with increasing pH, the affinity between the two proteins were also weakened. Under the conditions of the assembly reaction, ZipA formed aggregates and did not form filamentous polymers at any of the pH used (Fig. 6 vii–ix).

### Bis-ANS inhibited the binding of FtsZ and ZipA

**Figure 7:**
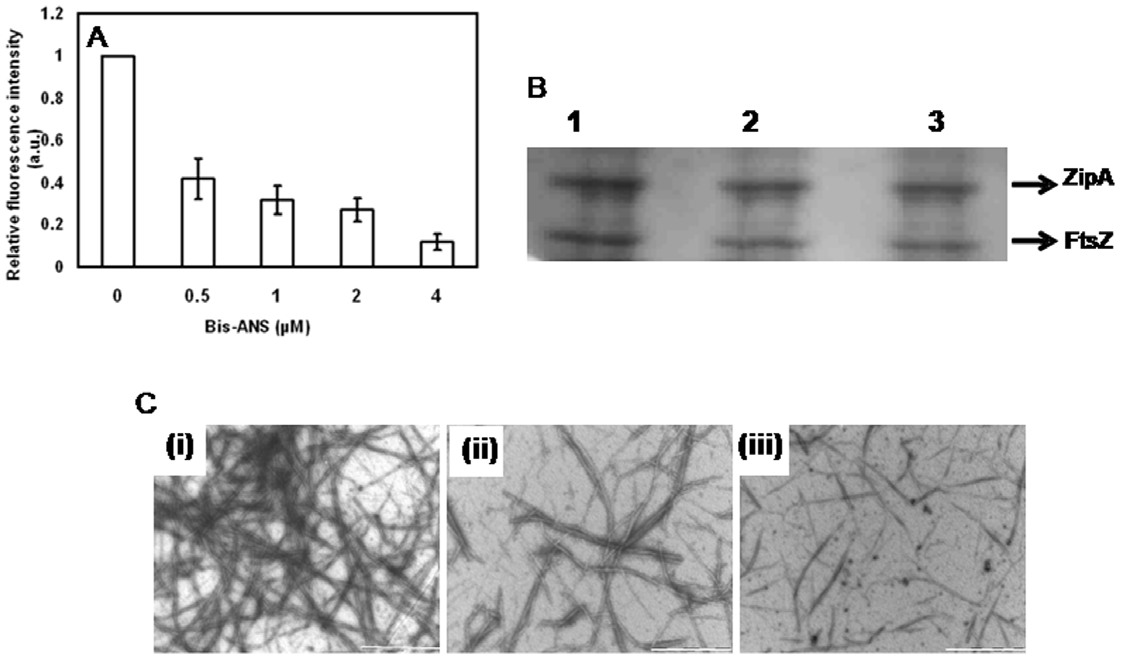
Bis-ANS inhibited the interaction of FtsZ and ZipA. Bis-ANS inhibited the binding of FITC-ZipA to FtsZ (Panel A). FITC-ZipA (0.5 µM) was incubated without or with different concentrations of bis-ANS for 5 min at 25°C. Then, 0.5 µM FtsZ was added to the reaction mixtures and incubated for an additional 15 min at 25°C and the fluorescence spectra were recorded. Bis-ANS inhibited the effects of ZipA on the assembly of FtsZ (Panel B). FtsZ (6 µM) was polymerized in the presence of 4 µM ZipA without or with different concentrations (5 and 10 µM) of bis-ANS. The polymeric FtsZ was collected by sedimentation and the amount of FtsZ in the pellets was estimated by coomassie-blue staining of the SDS-PAGE. The experiment was performed five times. Panel C shows electron micrographs of ZipA-induced FtsZ polymers in the absence (i) and presence of 5 µM (ii) and 10 µM (iii) bis-ANS, respectively. Scale bar is 1000 nm.

A hydrophobic probe bis-ANS has been widely used to determine the accessibility of hydrophobic surfaces [32], [33]. As described earlier (Fig. 4A), the interaction of FITC-ZipA with FtsZ was monitored by the increase of FITC-ZipA fluorescence upon binding to FtsZ. Bis-ANS reduced the enhancement of the fluorescence intensity of FITC-ZipA upon binding to FtsZ suggesting that it inhibited the interaction between FtsZ and FITC-ZipA (Fig. 7A). Further, bis-ANS was found to inhibit ZipA-promoted assembly of FtsZ (Fig. 7B & 7C). For example, bis-ANS (10 µM) reduced the amount of FtsZ pelleted by 28±4% as compared to that of the control (Fig. 7B). In addition, electron microscopic analysis showed that bis-ANS strongly reduced the number of FtsZ polymers suggesting that bis-ANS inhibited the ZipA-induced assembly and bundling of FtsZ protofilaments (Fig. 7C). The results together suggested that bis-ANS perturbed the interaction of ZipA and FtsZ.

## Discussion

In this study, FITC-ZipA was found to decorate FtsZ protofilaments along the length of FtsZ polymers suggesting that it copolymerized with FtsZ. ZipA suppressed the dilution-induced disassembly of FtsZ polymers suggesting that it stabilizes FtsZ protofilaments. The dissociation constants for FtsZ and ZipA interaction were determined as 43±20, 65±6 and 85±10 nM at pHs of 6.0, 6.8 and 8.0, respectively indicating that ZipA binds to FtsZ with a high affinity at all the three pHs examined (Fig. 4B). The results suggested that there could be tight binding between ZipA and FtsZ *in vivo* where the pH is in the range of 7.4–7.8. It was reported earlier that the assembly and bundling of FtsZ protofilaments decrease with increasing pH [19], [20]. ZipA was found to suppress the negative effect of increasing pH on the assembly of FtsZ to a certain degree (Fig. 6).

The presence of high salt (500 mM NaCl) did not reduce the binding affinity of ZipA to FtsZ instead it somewhat increased the binding affinity suggesting that hydrophobic interactions may play an important role in the binding of ZipA and FtsZ (Fig. 4C). We further explored the hydrophobic interaction between FtsZ and ZipA using a hydrophobic probe bis-ANS (Fig. 7). In agreement with the fluoresence spectroscopic analysis (Fig. 7A), bis-ANS was found to inhibit the amount of FtsZ polymerized in the presence of ZipA (Fig. 7B and 7C) suggesting that it reduced the interaction between ZipA and FtsZ. Using X-ray crystallograpy, it has been reported earlier that ZipA and FtsZ interact through their C-terminal tail regions (14). A 17 residue C-terminal tail peptide of FtsZ was found to make direct interatomic contacts with the C-terminal of ZipA and an analysis of the interacting residues revealed that the interaction was predominantly hydrophobic in nature. Seven amino acid residues (4 non polar and 3 polar) at the C-terminal tail of FtsZ were suggested to bind to 11 hydrophobic residues of ZipA, and spanned over all the six β strands of ZipA (14). It was suggested that 3 non-polar residues (Ile374, Phe377 and Leu378) on the FtsZ peptide tightly bound to ZipA [14] and were thought to be critical for the interaction of the two proteins. Further, alanine scanning analysis of the FtsZ peptide established the importance of the three above mentioned residues in its interaction with ZipA. Thus, hydrophobic interactions were expected to be dominant over the polar interactions, i.e., a Phe-Arg interaction and two hydrogen bonds [14]. The results presented in this study along with the previous findings indicated that the binding of ZipA and FtsZ involves hydrophobic interactions [14].

In summary, ZipA was found to stabilize the FtsZ protofilaments. It copolymerized with FtsZ and decorated FtsZ protofilaments along its length. ZipA bound to FtsZ with a high affinity at pH ranging from 6.0 to 8.0. Moreover, ZipA had positive influence on FtsZ assembly even under challenged assembly conditions at higher pHs. This can be directly correlated to the intracellular pH in *E. coli*, which is maintained in the range of 7.4–7.8. The bundling of FtsZ protofilaments supposed to strongly reduce at this pH was surmounted by the presence of ZipA. In addition, we have developed an equilibrium assay, which can be used to identify inhibitors of FtsZ-ZipA interaction.
